# Antibacterial Efficacy of Silver Nanoparticles on Endometritis Caused by *Prevotella melaninogenica* and *Arcanobacterum pyogenes* in Dairy Cattle

**DOI:** 10.3390/ijms19041210

**Published:** 2018-04-16

**Authors:** Sangiliyandi Gurunathan, Yun-Jung Choi, Jin-Hoi Kim

**Affiliations:** Department of Stem Cell and Regenerative Biotechnology, Konkuk University, Seoul 05029, Korea; yunjungc@konkuk.ac.kr

**Keywords:** antimicrobial therapy, endometritis, multiple drug-resistant bacteria, silver nanoparticles, oxidative stress

## Abstract

Bovine postpartum diseases remain one of the most significant and highly prevalent illnesses with negative effects on the productivity, survival, and welfare of dairy cows. Antibiotics are generally considered beneficial in the treatment of endometritis; however, frequent usage of each antibiotic drug is reason for the emergence of multidrug resistance (MDR) of the pathogenic microorganisms, representing a major impediment for the successful diagnosis and management of infectious diseases in both humans and animals. We synthesized silver nanoparticles (AgNPs) with an average size of 10 nm using the novel biomolecule apigenin as a reducing and stabilizing agent, and evaluated the efficacy of the AgNPs on the MDR pathogenic bacteria *Prevotella melaninogenica* and *Arcanobacterium pyogenes* isolated from uterine secretion samples. AgNPs inhibited cell viability and biofilm formation in a dose- and time-dependent manner. Moreover, the metabolic toxicity of the AgNPs was assessed through various cellular assays. The major toxic effect of cell death was caused by an increase in oxidative stress, as evidenced by the increased generation of reactive oxygen species (ROS), malondialdehyde, protein carbonyl content, and nitric oxide. The formation of ROS is considered to be the primary mechanism of bacterial death. Therefore, the biomolecule-mediated synthesis of AgNPs shows potential as an alternative antimicrobial therapy for bovine metritis and endometritis.

## 1. Introduction

Metritis and endometritis have a substantial influence on bovine health and productivity, with significant economic impacts to the dairy industry. Several studies have provided evidence that uterine infections are due to bacterial pathogenesis in the uterus [1,2,3,4,5,6,7]. The uterine infections caused by pathogenic bacteria lead to inflammation and infertility [3]. Uterine disease has unique and characteristic features, including a lower conception rate, along with increased intervals from calving to the first service or conception [8]. Endometritis is an inflammatory disease, which is associated with delayed uterine involution and poor reproductive performance [9]. Endometritis is frequently treated by intrauterine infusion of antibiotics [10]. However, the overloading and indiscriminate use of antibiotics for the treatment of uterine infections or any other microbial-related infections has led to the emergence of antibiotic-resistant strains. Indeed, the overwhelming usage of antibiotics has led to multidrug resistance (MDR), prolonged infection treatment, and increased mortality risk [11,12]. Furthermore, this increased risk of microbial resistance results in less effective conventional treatments. Therefore, it is necessary to overcome the limitations of the conventional continuous usage of antibiotics in the dairy industry and agriculture.

The application of nanoparticles has attracted huge interest in several fields, including biotechnology, biomedical sciences, and veterinary medicine. Several studies have explored the possibility of high-level nanotherapy in humans; however, the applications of nanotechnology in veterinary medicine have not reached the same level, and remain in a relatively innovative stage. Very recently, nanoparticles have been used as nutraceuticals, biocides, diagnostic tools, reproductive aids, and in drug and nutrient delivery in veterinary medicine [13], and show potential to serve as alternatives to conventional antimicrobial agents [11]. Thus, it is necessary to use nanotechnology to increase the safety of domestic animals, growth, production, and eliminate various diseases, so as to raise the income of farmers. Recently, the production of foods in the livestock industry using domestic animals has heavily relied on the use of antibiotics as growth promoters, leading to growing concern over the spread of microbial antibiotic resistance. The antibiotic resistance in bacteria leads, not only to a burden on public health, but also extends to the risk of therapy failure, along with subsequent economic impacts. Furthermore, the most severe consequence of antibiotic resistance is the threat of important endemic diseases in animals kept for food production [14]. Therefore, the development of innovative and cost-effective therapeutic strategies is in great demand for the treatment of veterinary animals. In this regard, nanoparticles appear to be suitable and alternative antimicrobial agents to overcome the alarming rate of the spread of antibiotics resistance, toward improved detection and killing of pathogenic bacteria. Recently, several studies have demonstrated plant and microbial extracts, essential oils, pure secondary metabolites, and newly synthesized molecules as potential antimicrobial agents [15,16,17].

Nanoparticle-based therapy is a promising approach to improve the balance between the efficacy and toxicity of systemic therapeutic intervention. Among the various metal nanoparticles available, silver nanoparticles (AgNPs) have attracted tremendous interest in biomedical applications, including for antimicrobial therapy, wound dressings, diagnosis and treatment, and contraceptive devices [18]. Moreover, AgNPs have been used as sensors, imaging, drug delivery, and for tissue engineering in veterinary medicine and animal production [19]. Thus, AgNPs represent a very promising therapeutic agent with unique potential against various microbial pathogens, with a particularly high capacity to effectively act on antibiotic-resistant bacteria [11,20]. To date, AgNPs have been widely used as effective antimicrobial agents against various bacteria, fungi, and viruses [21,22]. AgNPs can potentially inhibit multiple drug-resistant strains of *Staphylococcus aureus* and *Pseudomonas aeruginosa* that cause mastitis [20], and have proven to be effective against various antibiotic-resistant bacteria [12,21,22]. The mechanisms of inhibitory action of AgNPs are attributed to their high reactivity with bacterial proteins, sugars, and DNA, resulting in structural alterations to the cell wall and the membrane, ultimately leading to inhibition and cell death [23].

Therefore, developing a therapeutic strategy based on AgNPs to enhance the antibacterial effect represents a novel and promising approach [24], particularly in the era of multidrug resistance. Hence, in the present study, we synthesized and characterized AgNPs using the biomolecule apigenin as a reducing and stabilizing agent. Moreover, we isolated and characterized predominant isolates from clinical endometritis samples, and evaluated the effect of our developed biomolecule-assisted AgNPs against multiple drug-resistant Gram-negative and Gram-positive bacteria, including *Prevotella melaninogenica* and *Arcanobacterium pyogenes*. Finally, we evaluated the mechanism of toxicity of AgNPs in *P. melaninogenica* and *A. pyogenes.*

## 2. Results and Discussion

### 2.1. Synthesis and Characterization of AgNPs Using Apigenin

**Figure 1:**
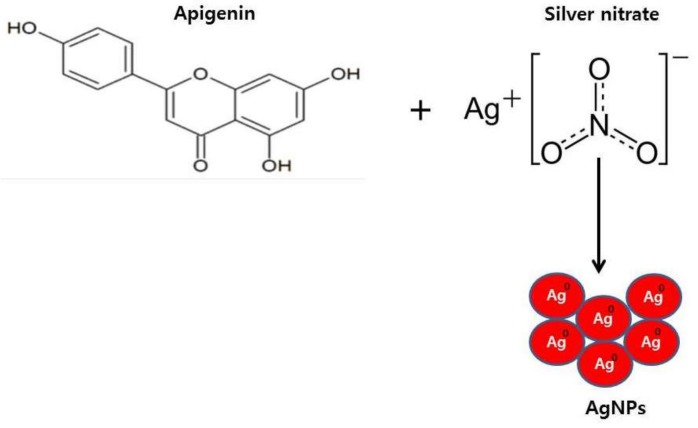
Schematic diagram of a simple and environmentally friendly approach for the synthesis of silver nanoparticles (AgNPs) by the reduction of silver nitrate to elemental silver using apigenin as a reducing agent.

Apigenin was reacted with AgNO_3_ at pH 8.0 and 40 °C for 6 h, and a yellow brown product was observed, indicating that apigenin could effectively reduce AgNO_3_ into AgNPs (Figure 1). 

**Figure 2:**
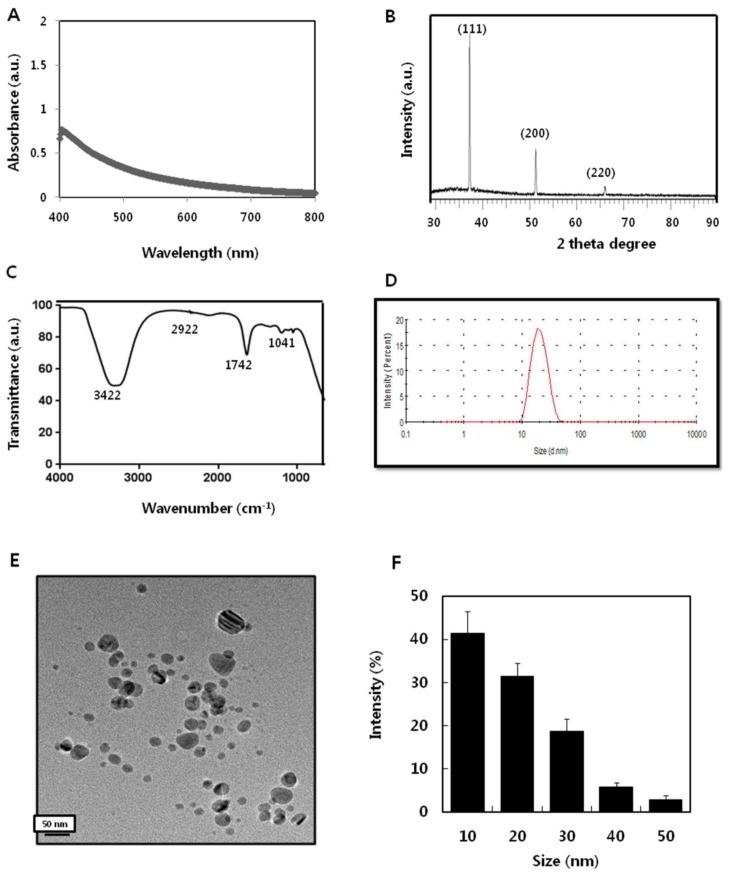
Synthesis and characterization of silver nanoparticles (AgNPs) using apigenin. (**A**) Absorption spectrum of AgNPs synthesized using apigenin. (**B**) X-ray diffraction spectra of AgNPs. (**C**) Fourier-transform infrared spectra of AgNPs. (**D**) Size distribution of AgNPs based on dynamic light scattering. (**E**) Transmission electron microscopy (TEM) images of AgNPs. (**F**) Histogram displaying predominant size of AgNPs.

The ultraviolet–visible spectra were used to determine the structure of the AgNPs based on their free surface electron plasmon oscillations. The shifting wavelength, like blue and red, reflect the size and shape of the AgNPs [25,26]. The absorption of AgNPs strongly depends on the particle size, dielectric medium, and chemical surroundings. Small spherical nanoparticles (<20 nm) exhibit a single surface plasmon band [25,26]. The synthesized particles exhibited maximum absorption at 407 nm, which represents the characteristic peak for AgNPs (Figure 2A). In line with these findings, several studies have demonstrated that flavonoids and phenolic compounds, such as quercetin and caffeic acid, can reduce Ag^+^ quickly, and can act effectively as both reducing and stabilizing agents [23,27,28].

The X-ray diffraction pattern of the AgNPs synthesized by apigenin is shown in Figure 2B. Several strong Bragg reflections were observed, corresponding to the (111), (200), and (220) reflections of face centred cubic (fcc) silver [29]. The high-intensity peak of Ag (111) was observed in the sample, which indicated the crystalline nature of the particles. The diffraction peaks of the synthesized AgNPs peaks were very sharp, and clearly suggested that the AgNPs synthesized using apigenin are crystalline in nature [30]. The synthesized particles were crystalline, and the size was determined to be 10 nm using the Debye–Scherrer formula. According to the Debye–Scherrer formula, the peak position (111) reflects that the dimensions of the particles are 10 nm [31].

Fourier-transform infrared (FTIR) measurement was carried out to confirm the involvement of various functional groups for reduction of Ag^+^ in apigenin, resulting in the capping/stabilization of AgNPs. The FTIR spectrum showed absorption bands at 3422, 2922, 1742, and 1042 cm^−1^, indicating the presence of a capping agent within the nanoparticles (Figure 2C). The band at 3422 cm^−1^ in the spectrum corresponds to the O–H stretching vibration indicating the presence of alcohol and phenol [28]. Bands at the 2922 cm^−1^ region were observed, arising from C–H stretching of aromatic compound. The band at 1743 cm^−1^ was assigned to C–C stretching. Several studies have reported that functional groups such as alcohol, phenol, and amines play a role in the stability/capping of AgNPs [32]. The bands at 1042 cm^−1^ were assigned to N–H and C–N stretching vibrations of the proteins, respectively [33]. Collectively, the FTIR data confirmed that various functional groups found in apigenin facilitate the capping and stabilization of AgNPs.

Next, dynamic light scattering (DLS) was performed to determine the size distribution of colloidal AgNPs in the range of 1–100 nm. The DLS method is widely used in studies dealing with the synthesis, functionalization, and biomedical use of nanoparticles that exhibit plasmon resonance, primarily with regard to gold and silver nanoparticles [34]. The synthesized particles showed an average size of 20 nm, which is the hydrodynamic size of an entire complex, rather than the geometrical size of a particle itself (Figure 2D). In general, the dispersion of the DLS particle number size distributions noticeably exceed the size dispersion obtained from transmission electron microscopy (TEM) images [34]. We further confirmed the particle size by TEM, which is a more reliable method for determining the size and shape of particles. The TEM images revealed that most of the particles are significantly spherical in shape with a size of 10 nm (Figure 2E). The histogram of TEM images determined from the calculation of several particles demonstrated that the TEM data on the particle number and size were apparently accurate, exhibiting the presence of a noticeable number of particles with diameters of 10 nm (Figure 2F). Thus, the data derived from DLS and TEM revealed that the most predominant size of the particles was 10 nm.

### 2.2. Isolation, Identification, and Characterization of Bacteria from Endometritis Samples

Bacterial isolates obtained from endometritis samples were cultured, identified, and characterized as described in the Material and Methods. Among 40 swabs, 20 were found to be bacteriologically positive by characterization of bacteria both phenotypically and biochemically [35,36]. The most frequently isolated bacterium was *Prevotella melaninogenica* (30%), followed by *Arcanobacterium pyogenes* (25%), *Escherichia coli* (20%), *Streptococcus* spp. (15%), *Staphylococcus* spp. (10%), *Campylobacter fetus* (8%), *Klebsiella* spp. (5%), *P. aeruginosa* (3%), and *Clostridium* spp. (1%). Similarly, Udhayavel et al. [37] reported that out of 30 samples evaluated, 25 exhibited different strains of bacteria, including *E. coli* (36.66%), *Klebsiella* spp. (30%), *Proteus* spp. (13.33%), *P. aeruginosa* (6.66%), and *Clostridium* spp. (3.33%). Sharma et al. [36] reported that the most frequently identified bacteria isolates from uterine discharge samples included *E. coli* (32.26%), *Bacillus cereus* (22.58%), *S. aureus* (16.13%), and mixed cultures of *B. cereus* and *S. aureus* (9.68%), and *E. coli* and *Proteus vulgaris* (3.23%). Altogether, our data agree with previous findings and indicate that *P. melaninogenica* and *A. pyogenes* represent the most dominant bacterial isolates found in endometritis clinical samples from the district of Coimbatore, Tamil Nadu.

### 2.3. Isolation of MDR

The antibiotic susceptibility test was performed according to Clinical and Laboratory Standards Institute (CLSI) procedures. We selected more profound isolates for further antibiotic susceptibility testing. Among the several isolates tested, MDR isolates were defined as those showing resistance or intermediate susceptibility to more than three antimicrobials. The result of antimicrobial tests showed that all of the isolates of *P. melaninogenica* were resistant to ampicillin (90.0%), cefalotin (79.0%), sulfamethoxazole/trimethoprim (65.2%), ciprofloxacin (54.6%), oxolinic acid (45.4%), gentamicin (43.8%), chloramphenicol (40.0%), cefotaxime (23.8%), ceftazidime (18.8%), amoxicillin/clavulanic acid (10.0%), and aztreonam (5.0%). The *A. pyogenes* isolates exhibited resistance to all of the antimicrobial agents tested, with particularly high levels of resistance found to chloramphenicol (100%), amoxicillin (86.9%), ampicillin (76.1%), florfenicol (69.7%), penicillin (66.1%), oxytetracycline (64.2%), and tetracycline (50%). Thus, the results from antibiotic susceptibility tests showed that *P. melaninogenica* and *A. pyogenes* were resistant to at least three of the antimicrobial agents tested, indicating that these are MDR isolates. Further experiments were carried out in *P. melaninogenica* and *A. pyogenes* to evaluate the impact of AgNPs on MDR bacteria in endometritis.

### 2.4. Minimum Inhibitory Concentration (MIC) and Minimum Bactericidal Concentration (MBC) of AgNPs

The MIC is the lowest concentration of AgNPs that will inhibit the visible growth of a microorganism after overnight incubation. The MIC was determined in brain heart infusion (BHI) broth using serial two-fold dilutions of AgNPs in concentrations ranging from 0.1 μg/mL to 1.0 μg/mL, with an adjusted bacterial concentration of 1 × 10^8^ colony forming units (cfu)/mL (0.5 McFarland’s standard). Medium without AgNPs was used as a control. The results from the cell viability assay suggested that AgNPs inhibit bacteria in a dose-dependent manner, and the MIC values of AgNPs against *P. melaninogenica* and *A. pyogenes* were found to be 0.8 and 1.0 μg/mL, respectively. The MBC is the lowest concentration of AgNPs required to kill a particular bacterial strain. The MBC values of AgNPs against *P. melaninogenica* and *A. pyogenes* were found to be 1.0 and 1.5 μg/mL, respectively. As the concentration of AgNPs increased to the level of the MIC of the respective strains, no growth was observed. The bactericidal effect of the AgNPs was dependent on several factors, such as the concentration of AgNPs, size, shape, physicochemical properties, and the initial bacterial concentration. In general, AgNPs showed better antimicrobial activity against the Gram-negative bacterium *P. melaninogenica* when compared to that against the Gram-positive bacterium *A. pyogenes*. Our findings are consistent with previous reports suggesting that Gram-positive bacteria are less susceptible to the antimicrobial activity of silver [24,38,39].

### 2.5. Dose- and Time-Dependent Effect of AgNPs on Cell Viability of P. melaninogenica and A. pyogenes

**Figure 3:**
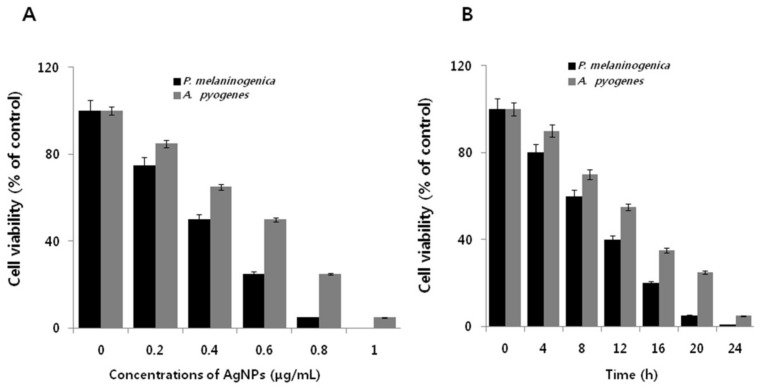
Antibacterial activity of AgNPs on *P. melaninogenica* and *A. pyogenes.* (**A**) *P. melaninogenica* and *A. pyogenes* were incubated with various concentrations of AgNPs. Bacterial cell survival was determined at 24 h based on a CFU count assay. (**B**) *P. melaninogenica* and *A. pyogenes* cells were incubated with 0.8 and 1.0 μg/mL of AgNPs, respectively, for 24 h.

To further promote the use of AgNPs in nanomedicine to overcome MDR in Gram-positive and Gram-negative bacteria, the dose-dependent effect of AgNPs was assessed in *P. melaninogenica* and *A. pyogenes* to determine their relative susceptibilities to AgNPs, and the extent of bactericidal activity. Figure 3A shows the potential toxic effect of apigenin-assisted AgNPs on *P. melaninogenica* and *A. pyogenes.* The bacterial strains were treated with various concentrations (0.2–1 μg/mL) of the 10 nm AgNPs. The results showed a dose dependent effect on cell viability compared to the negative control. Furthermore, cell viability decreased with increasing AgNPs concentrations. No visible growth was observed at their respective MIC values (0.8 and 1.0 μg/mL) in *P. melaninogenica* and *A. pyogenes.* In the case of *P. melaninogenica*, the introduction of 0.8 μg/mL of AgNPs reduced bacterial viability by approximately 95%, as compared to that of the control sample. Furthermore, increasing the concentration of AgNPs to 1 μg/mL inhibited bacterial growth dramatically with no visible growth observed, whereas introduction of a similar concentration of AgNPs (i.e., 0.75 μg/mL) reduced cell viability by approximately 75% as compared to the control sample. However, the higher concentrations of 0.75 and 1.0 μg/mL rapidly inhibited the growth of bacteria (Figure 3A,B).

We previously reported that the antibacterial activity of AgNPs with an average size of 10 nm produced from the cellular extract of *Bacillus cereus* required a 10-fold higher concentration to exhibit a similar antibacterial effect against *Escherichia fergusonii* and *Streptococcus mutans,* which is due to the type of reducing agents and type of bacteria [40]. For instance, AgNPs coated with lipoic acid and polyethylene glycol exhibited lower cytotoxicity as compared with AgNPs coated with tannic in a gingival fibroblast model [41]. Strydom et al. [42] suggest that modification of silver sulfadiazine using dendrimers displayed potential antibacterial activity. The antimicrobial activity of AgNPs also depends on the surface area, which effectively interacts with a certain microorganism. Several studies have substantiated that a unique feature of large surface area of nanoparticles have the significant possibility interact with microbes [21,43].

### 2.6. Dose- and Time-Dependent Effect of AgNPs on the Biofilm Activity of P. melaninogenica and A. pyogenes

**Figure 4:**
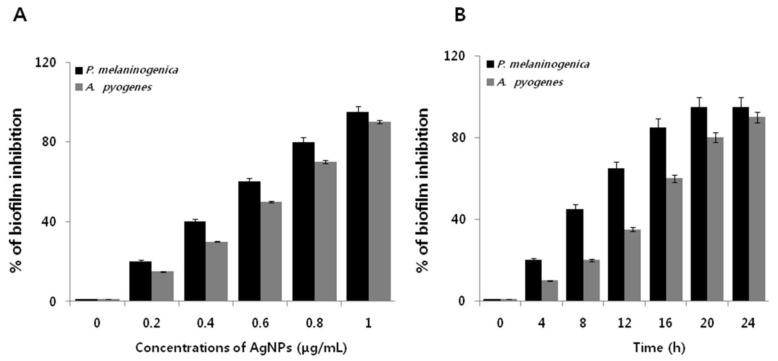
Anti-biofilm activity of AgNPs on *P. melaninogenica* and *A. pyogenes.* (**A**) *P. melaninogenica* and *A. pyogenes* were incubated with various concentrations of AgNPs. Anti-biofilm activity was measured using 96-well flat-bottom polystyrene tissue culture plates. (**B**) *P. melaninogenica* and *A. pyogenes* cells were incubated with 0.8 and 1.0 μg/mL of AgNPs respectively for 24 h.

To examine the anti-biofilm activity of AgNPs on *P. melaninogenica* and *A. pyogenes,* the bacteria were grown in tissue culture plates in the presence and absence of AgNPs for 24 h. Both bacterial strains were grown for 24 h in microtiter plate wells and then treated with 0.1–1.0 μg/ mL AgNPs (Figure 4A). AgNPs decreased the biofilm activity of *P. melaninogenica* and *A. pyogenes* by more than 95% and 90%, respectively. Our findings are consistent with previous reports in various Gram-negative and Gram-positive bacteria. Interestingly, AgNPs inhibited biofilm formation faster within 20 h in *P. melaninogenica* than in *A. pyogenes,* which is likely due to the structural nature of the cell wall and membrane (Figure 4B). Bacteria biofilms are resistant to antibiotics, disinfectants, and components of the innate and adaptive inflammatory responses [26,44]. AgNPs potentially inhibit cell viability and biofilm formation against *P. aeruginosa* and *Staphylococcus epidermidis* by inhibiting production of exopolysaccharides, which are essential for biofilm formation [25,45]. Plant extract-mediated synthesis of AgNPs efficiently inhibited biofilm formation in *Helicobacter pylori* and *Helicobacter felis* [38]. Martinez-Gutierrez et al. [46] demonstrated the quorum-quenching activity of AgNPs against various Gram-negative and Gram-positive bacteria. Taken together, our results suggest that the apigenin-mediated synthesis of AgNPs could be a potential and viable alternative anti-biofilm agent.

### 2.7. AgNPs Induce Metabolic Toxicity in P. melaninogenica and A. pyogenes

**Figure 5:**
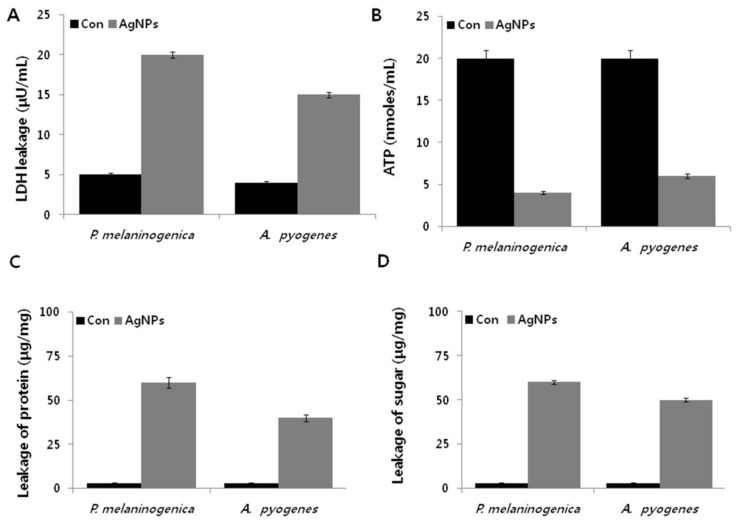
Metabolic cytotoxicity of AgNPs on *P. melaninogenica* and *A. pyogenes. P. melaninogenica* and *A. pyogenes* cells were incubated with 0.8 and 1.0 μg/mL of AgNPs respectively for 12 h, and the (**A**) LDH activity, (**B**) ATP levels, (**C**) protein levels, and (**D**) sugar levels were determined.

Perturbations of metabolic activity are a possible strategy to impact the efficacy of antimicrobial therapy. Lactate is a very important end product of carbohydrates synthesis in bacteria. To evaluate the effect of AgNPs on oxidative stress-induced metabolic changes, a lactate dehydrogenase (LDH) assay was performed in cells exposed to AgNPs for 12 h [47]. As shown in Figure 5A, the level of LDH in *P. melaninogenica* and *A. pyogenes* was four-fold higher than that of the control group. Although both bacteria exhibited similar levels of LDH, that of the Gram-positive bacterium *A. pyogenes* was slightly lower than that of the Gram-negative bacterium *P. melaninogenica*, which is due to the architecture of the cell wall and membrane. Our results clearly demonstrated that the activities of respiratory chain dehydrogenases (RCD) in both *P. melaninogenica* and *A. pyogenes* were inhibited by AgNPs, which is in line with previous studies demonstrating the mechanism of antimicrobial action [23,48,49,50,51]. One possible mechanism underlying this metabolic disturbance is the entry of AgNPs into the cells to RCD and alter dissolved oxygen levels in culture [45]. Another potential mechanism is that the Ag^+^ of the AgNPs interact with the thiol (–SH) group of cysteine [51].

Using in silico genome-scale metabolic models, Brynildsen et al. [52] clearly demonstrated that an increase in the intracellular production of endogenous reactive oxygen species (ROS) could agitate the production and usage of ATP. ATP is an energy-rich molecule that governs various cellular functions such as survival, growth, and replication, and acts as a major signaling molecule [53]. In line with that prediction, we sought to determine the level of ATP in AgNPs-treated *P. melaninogenica* and *A. pyogenes.* The level of ATP in AgNPs-treated samples was significantly lower by up to five-fold compared to that of the control samples (Figure 5B), indicating that AgNP-induced cellular stress significantly affects ATP synthesis in *P. melaninogenica* and *A. pyogenes*, which is a critical factor for bacterial growth and reproduction [25]. AgNPs directly affect FOF1-ATPase activity and H^+^-coupled transport [54]. FOF1-ATPase plays a crucial role in cell metabolic processes, including bacterial growth, metabolic regulation, and cell survival. Therefore, the data from the present study and total body of previous work suggest that metabolic activity contributing to ATP production is an integral part of the bactericidal toxicity of AgNPs.

To validate the effect of AgNPs on the weakening of metabolic activity, we further explored the levels of proteins and sugars. We previously demonstrated that AgNPs are potential agents to increase protein leakage by altering the membrane permeability in bacteria [40]. *P. melaninogenica* and *A. pyogenes* were treated with 0.8 and 1.0 μg/mL of AgNPs, respectively, and the amount of protein released in the suspension was estimated using the Bradford assay. The results showed that AgNPs remarkably increased the leakage of proteins compared to the control group (Figure 5C). However, the leakage from *P. melaninogenica* cells treated with AgNPs was significantly higher (60 μg/mg) than that of *A. pyogenes* (40 μg/mg), suggesting that the antibacterial sensitivity of Gram-negative bacteria is much stronger than that of Gram-positive bacteria. Similarly, Kim et al. [51] and Gurunathan et al. [24] found that the leakage of proteins was significantly higher in Gram-negative bacteria than in Gram-positive bacteria with 60 and 50 μg of reducing sugars leaking from *P. melaninogenica* and *A. pyogenes* treated with AgNPs, respectively (Figure 5D). A previous study showed that after *E. coli* cells were exposed to AgNPs (10 μg/mL) for 2 h, up to 102.5 μg per bacterial dry weight of 1 mg of reducing sugars leaked out of the cells [49]. This differential leakage amount could be due to the structural features of the cell wall of *A. pyogenes*, which is essential for protecting the bacteria various toxic agents [23,24,51]. The impairment of the function of LDH could lead to increased leakage of proteins and other macromolecules. Altogether, all of the available evidence from various bacteria clearly indicate that AgNPs could alter membrane permeability and eventually damage the structure of the bacteria cell membrane by osmotic imbalance, resulting in the leakage of macromolecules such as proteins and reducing sugars, leading to the death of bacteria. This mechanism highlights the significant potential of the antibacterial activity of AgNPs.

### 2.8. AgNPs Induce Cellular Toxicity and Oxidative Stress in P. melaninogenica and A. pyogenes

**Figure 6:**
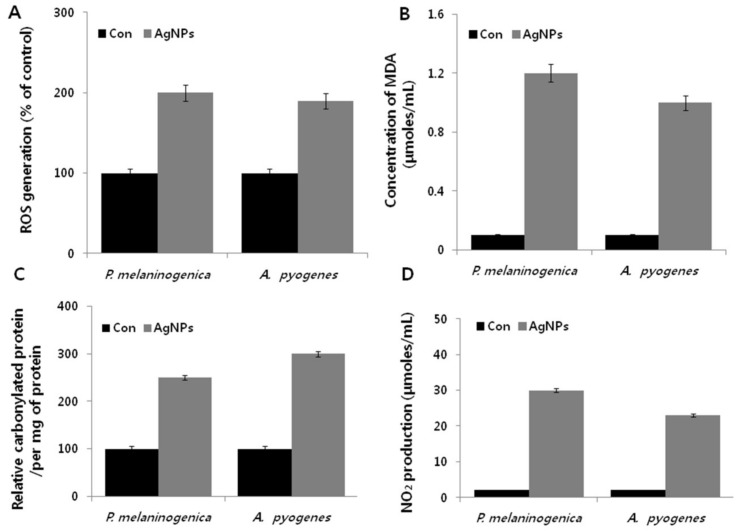
Effect of AgNPs on cellular toxicity in *P. melaninogenica* and *A. pyogenes.* (**A**) *P. melaninogenica* and *A. pyogenes* cells were treated with 0.8 and 1.0 μg/mL of AgNPs respectively for 12 h. ROS generation was measured using DCFDA. (**B**) MDA levels were measured using a TBARS assay. (**C**) The relative protein carbonyl content was evaluated compared to the total protein content. (**D**) The quantity of NO was quantified spectrophotometrically using the Griess reagent.

To understand the effects of AgNPs on cell viability and metabolic toxicity, we further examined how the influence of AgNPs on bacterial metabolism could offer insight into their mechanisms of action, leading to enhanced therapeutic approaches for both humans and animals. Major classes of bactericidal antibiotics induce cell death in bacteria by stimulating the production of highly deleterious hydroxyl radicals [55]. A similar mechanism has been demonstrated for AgNP-induced cell death in a variety of bacteria, including the most representative Gram-negative and Gram-positive bacteria, such as *P. aeruginosa*, *Shigella flexneri*, *S. aureus*, and *Streptococcus pneumonia* [24]. However, to our knowledge, no study has demonstrated the mechanism of AgNPs on the oxidative stress-induced cell death in *P. melaninogenica* and *A. pyogenes.* The bacteria were treated with the respective MIC of the AgNP, and ROS generation was measured using the 2′,7′-dichlorofluorescin diacetate (DCFDA) assay. The results indicated that AgNPs induced two-fold higher levels of ROS in *P. melaninogenica* and *A. pyogenes* compared to the control (Figure 6A). An increased level of ROS leads to an imbalance between pro-oxidants and antioxidants, which causes failure in normal physiological redox-regulated functions [55]. Indeed, ROS can be induced by various external sources, such as chemicals, antibiotics, nanoparticles, and cold and heat stress, consequently leading to loss of cell viability [33,40,55,56,57].

Next, we examined the level of malondialdehyde (MDA), which is a well-known marker in eukaryotic cells for oxidative stress, as it is generated from lipids by stimulation of oxidative stress. To ascertain the MDA levels in AgNP-treated bacteria, we used thiobarbituric acid. Treatment with AgNPs led to increased levels of MDA by several fold in *P. melaninogenica*, compared to the control group (Figure 6B); similar increases were also observed in *A. pyogenes*. These findings suggest that lipid oxidation induced MDA production in bacteria. Belenky et al. [58] found that antibiotic-treated *E. coli* cells exhibited cytotoxic changes that were indicative of oxidative stress, including higher levels of protein carbonylation. The carbonylation of proteins could lead to protein dysfunction [55,56,57]. Therefore, we hypothesized that AgNPs could target the well-known oxidative stress biomarker of carbonylation. To measure the carbonyl content, *P. melaninogenica* and *A. pyogenes* were treated with AgNPs for 12 h, which led to significant increases in protein carbonylation, up to 12 times above that of the control (Figure 6C). These findings are consistent with the effects of bacteria treated with Ampicillin (Amp), kanamycin (Kan), or Nor [58]. Nitric oxide (NO) produced by bacterial nitric oxide synthase (NOS) acts as a cytoprotective agent against oxidative stress in *S. aureus, Bacillus anthracis,* and *Bacillus subtilis* [58]. To explore whether AgNPs induce the production of NO or inhibit, we examined the effect of NO production in AgNP-treated *P. melaninogenica* and *A. pyogenes.* AgNPs induced the production of NO in both *P. melaninogenica* and *A. pyogenes* (Figure 6D). Interestingly, NO production was significantly higher in the Gram-negative bacterium than in the Gram-positive bacterium, which indicates that *P. melaninogenica* may be more immune to the stress created by AgNPs. Previous studies demonstrated that the oxidative stress generated by AgNPs was associated with reduction in the levels of reactive nitrogen intermediates in bacteria treated with different antibiotics [59]. Collectively, the present study suggests that AgNPs interact with bacterial cells via the cell wall and membrane, resulting in the production of free radicals to, in turn, induce oxidative stress and cause various dysfunctions to macromolecules, including lipids, proteins, and nucleic acids [22,60,61,62,63].

### 2.9. Effect of AgNPs on the Expression of Antioxidative Markers in P. melaninogenica and A. pyogenes

**Figure 7:**
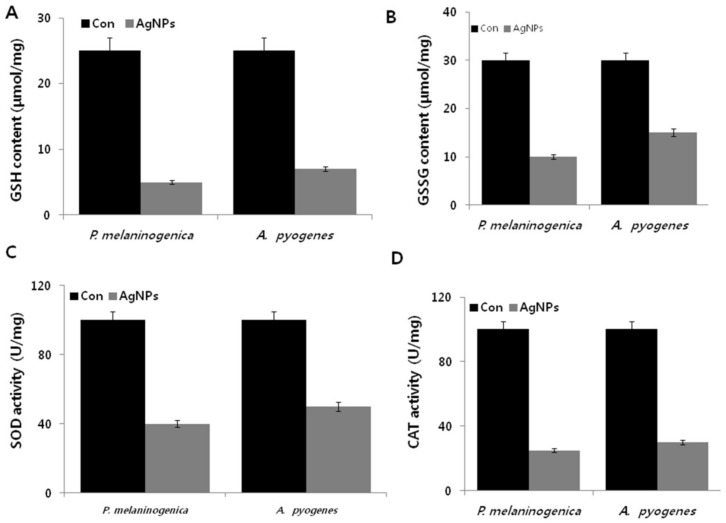
Effect of AgNPs on antioxidants. *P. melaninogenica* and *A. pyogenes* cells were treated with 0.8 and 1.0 μg/mL of AgNPs respectively for 12 h, and the (**A**) GSH levels, (**B**) GSSG levels, (**C**) superoxide dismutase (SOD) activity, and (**D**) catalase (CAT) activity were measured as described in the Materials and Methods.

The antioxidative stress response counteracts the effect of pro-oxidants to maintain normal physiological redox-regulated functions. Masip et al. [64] demonstrated that a depressed ratio of reduced glutathione (GSH) to oxidized glutathione (GSSG) is considered an indicator of oxidative stress. Thus, the levels of GSH and GSSG were determined in *P. melaninogenica* and *A. pyogenes* treated with AgNPs for 12 h, demonstrating decreased levels of GSH coupled with highly significant decreases in GSSG (Figure 7A,B). This decreased level of GSH in the AgNP-treated cells suggests an inability to protect the cells from oxidative stress, so that the cells were subjected to cell death due to overwhelming oxidative stress. Banerjee et al. [65] observed increased levels of oxidative stress and decreased levels of antioxidants in *E. coli* cells treated with an iodinated chitosan–silver nanoparticle composite. Similarly, *E. coli* and *P. aeruginosa* treated with AgNPs exhibited a similar trend [20,63]. Together, these changes in metabolite levels suggest that decreased GSH is unable to compensate for the ongoing turnover and consumption by pro-oxidant activities. Collectively, these data suggest that the complex metabolic changes of AgNPs are induced by oxidative stress. 

Silver ions are responsible for the formation of free radicals. Therefore, we next examined enzymes with antioxidant effects such as superoxide dismutase (SOD) and catalase in *P. melaninogenica* and *A. pyogenes* treated with 0.8 and 1.0 μg/mL of AgNPs for 12 h. AgNPs decreased the level of SOD by up to six- and five-fold in *P. melaninogenica* and *A. pyogenes*, respectively (Figure 7C). Similarly, the catalase activity sharply decreased, which was slightly higher than the SOD activity (Figure 7D). These results indicate that AgNP treatment might decrease the antioxidant levels in *P. melaninogenica* and *A. pyogenes.* Nanoparticle-mediated ROS generation has been shown to disrupt the electron transport assemblies of the plasma membrane and regulate various antioxidant enzymes, including NADPH-dependent flavoenzyme, catalase, glutathione peroxidase, and SOS [64]. For example, *Pseudomonas putida* exposed to AgNPs showed increased lipid peroxidation with a simultaneous decrease in the antioxidant defense system [65]. AgNPs potentially induce the alteration of major macromolecules in bacteria by an alarming rate of increase of ROS in bacterial cells [66]. Our study suggests that AgNP-induced oxidative stress could disturb the stability of the cell membrane and increase the membrane permeability to release various membrane-bound enzymes.

### 2.10. Antibiotics Induce DNA and RNA Oxidation in P. melaninogenica and A. pyogenes

**Figure 8:**
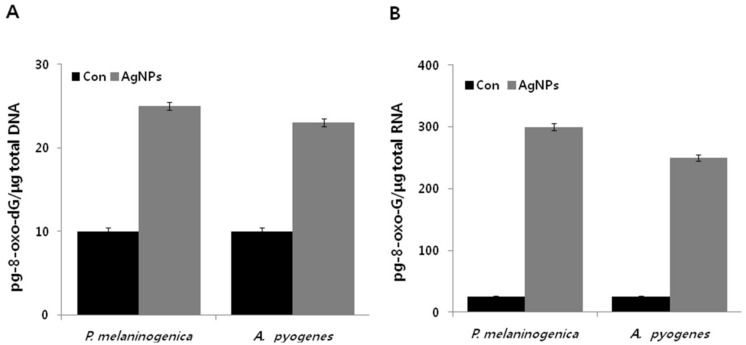
Measurement of DNA/RNA oxidation. *P. melaninogenica* and *A. pyogenes* cells were treated with 0.8 and 1.0 μg/mL of AgNPs respectively for 12 h, and oxidation of (**A**) DNA was measured by 8-oxo-dG levels and (**B**) oxidation of RNA was measured by 8-oxo-G levels.

The modification of DNA and RNA is a core consequence of oxidative stress, and the oxidation of guanine is due to low redox potential [67]. 8-Oxo-7,8-dihydroguanine (8-oxo-dG) is considered to be a biomarker of oxidative stress in DNA or RNA, which is merely oxidation at the level of the nucleoside triphosphate [55]. Oxidation of DNA leads to mismatched base-pairing, mutagenesis, and DNA double-strand breaks [68,69,70]. Similarly, oxidation of RNA causes protein mistranslation, protein aggregation, and resulting cellular damage [71]. To quantify the levels of both 8-Oxo-2′-deoxyguanosine (8-oxo-Dg) and 8-Oxo-2′-oxyguanosine (8-oxo-g), we utilized an enzyme-linked immunoassay (ELISA)-based technique. The results showed that the levels of 8-oxo-dG in the DNA pool significantly increased by up to two to three-fold upon the addition of AgNPs, compared to those of untreated cells (Figure 8A). Interestingly, the levels of 8-oxo-G were more dramatically increased, by up to 12-fold. These findings suggest that the basal levels of 8-oxo-G on the RNA pool were significantly elevated by approximately more than 12 times the levels of 8-oxo-dG on DNA (Figure 8B). Our results are consistent with prior work in cells treated with antibiotics such as Amp, Kan, and Nor [55]. Similarly, *E. coli* cells treated with AgNPs displayed a two-fold higher level of 8-oxo-dG on DNA compared to the control [66]. Similar results were also observed with polymeric nanoparticles that induce DNA damage caused by elevation of ROS level [72,73,74,75,76,77,78]. Our findings are consistent with mechanisms of other antimicrobial agents such as antibiotics, and suggest that AgNPs induce a toxic effect on bacterial structures by oxidative mechanisms, leading to the oxidation of macromolecules such as lipids, DNA, and proteins, ultimately resulting in bacterial death [22,52,55].

## 3. Materials and Methods

### 3.1. Materials

BacTiter-Glo™ Microbial Cell Viability Assay Reagent was purchased from Promega (Madison, WI, USA). All other chemicals were purchased from Sigma-Aldrich (St. Louis, MO, USA) unless otherwise stated.

### 3.2. Synthesis and Characterization of AgNPs

AgNP synthesis was performed using apigenin according to a previously described method [20]. First, 1.0 mg/mL apigenin was dissolved in dimethyl sulfoxide, AgNO_3_ was dissolved in water at a concentration 5 mM, and finally, both apigenin and AgNO_3_ were incubated at 40 °C for 6 h. Further characterization of the synthesized AgNPs was performed as previously described [73].

### 3.3. Sample Collection

Forty uterine secretion samples were collected from Holstein and Jersey cows from clinical cases of endometritis. The samples were collected in sterile containers. Prior to bacteriological examination, the samples were mixed with sterile distilled water in a vortex shaker for 10–15 min, and divided into two halves. One half was used for bacterial counting, and the other half was used for identification of organisms after direct inoculation on different agar plates, including BHI, nutrient, blood, and MacConkey (Sigma-Aldrich, St. Loius, MO, USA) agar plates.

### 3.4. Bacterial Characterization

Isolation of bacteria from the uterine discharge was performed by directly streaking it and plating directly onto appropriate aerobic and anaerobic media, such as blood agar/MacConkey agar/BHI agar and eosin methylene blue (EMB) plates, to obtain discrete colonies; different types of agar media were used to distinguish various types of microorganisms, such as Gram-negative and Gram-positive bacteria. The plates were incubated at 37 °C aerobically, and examined for the presence of bacteriological growth after 24–72 h of incubation. Single isolated colonies of each purified isolate were inoculated in nutrient broth tubes, and incubated at 37 °C for 48 h to obtain broth cultures for biochemical testing. Sampling, growth, and characterization of bacteria were performed as described previously [20,74]. Each sample (0.5 mL) was cultured on blood agar/MacConkey agar/BHI agar and EMB using the spreading technique, and the plates were incubated at 37 °C for 24 h. Bacterial colonies were identified using Bergey’s Manual for Determinative Bacteriology and 16S RNA sequencing [74]. The bacterial load was determined by counting the CFUs formed on the agar plates after 24 h. After inoculation, the plates were incubated aerobically for 24–72 h at 37 °C. 

### 3.5. Bacterial Strains and Growth Conditions 

Bacterial growth and media preparation were performed according to the type of bacteria [25]. In brief, all cultures were first grown aerobically at 37 °C in BHI/Mueller–Hinton broth (MHB) media. Cultures were maintained by streaking a bacterial colony in MHB agar plates and subculturing every fortnight. Pure colonies were isolated and stored at −80 °C. All test strains were grown and maintained in BHI/MHB medium. Cells were grown and harvested by centrifugation at 6000 rpm for 10 min and then resuspended in sterile MHB medium until reaching an optical density at 600 nm (OD_600_) of 1.0.

### 3.6. MIC and MBC Determination

AgNP susceptibility tests were carried out in 96-well microtiter plates using a standard two-fold broth microdilution of the antibacterial agents in BHI/MHB following the CLSI guidelines (CLSI, 2003). To determine the MICs of AgNPs, all test strains were exposed to 0–100 µg/mL AgNPs. AgNPs solutions were prepared using phosphate-buffered saline (PBS) and tested for antibacterial efficacy. The appropriate AgNP concentration and 1 mL of the bacterial suspension were mixed in MHB medium to obtain a final bacterial concentration of 10^5^–10^6^ CFUs/mL and incubated for 24 h. After treatment, 100 μL of the reaction mixture was diluted to 1 mL, and 100 μL of the total mixture was used for plating. Loss of cell viability was determined using the colony counting method. Colonies were counted and compared with the number of colonies on the control plates to calculate changes in cell growth. BHI/MHB medium without AgNP-based materials was used as the control. All treatments were prepared in triplicate, and repeated in at least three independent experiments. The MIC was considered as the lowest concentration that visibly inhibited bacterial growth. The MBC was considered to be the lowest concentration of an antibacterial agent required to kill a bacterium. Control tests were carried out with solutions containing all of the reaction components without the addition of AgNPs.

### 3.7. Isolation of MDR Bacteria

Antimicrobial resistance patterns were determined by the Kirby–Bauer disk diffusion test based on a method described by Santos et al. [79]. We used the following antibiotics in this test: amikacin, norfloxacin, chloramphenicol, gentamicin, sulfamethoxazole/trimetoprim, ofloxacin, cefotaxime, ceftriaxone sodium, amoxicillin, ampicillin, ceftiofur, florfenicol, oxytetracycline, penicillin, spectinomycin, streptomycin, imipenem, piperacillin/tazobactam, ampicillin/sulbactam, metronidazole, and tetracycline. The MDR rate was calculated by dividing by the total number of antimicrobial resistant groups. The inhibition zone diameter around each disk was measured, and was interpreted as described in the NCCLS (2013). Bacteria exhibiting resistance to more than two classes of antibiotics were checked for sensitivity against different combinations of antibiotics. 

### 3.8. Antimicrobial Activity of AgNPs 

Assessment of the microbial toxicity of AgNPs was performed as previously described [12,24]. In brief, to examine the effects of AgNPs on growth of the isolates, overnight cultures were centrifuged at 6000 rpm for 5 min, washed with 1× PBS, and the pellet was resuspended in saline buffer. Finally, the OD_600_ of the sample was adjusted to 0.1. Cells (5 × 10^5^ cells in 96-well round-bottom plates in triplicate) were exposed to different concentrations of AgNPs. Bacteria were harvested at the indicated time points or dose responses, and the number of cfus was counted. Media only and media containing AgNPs only were used as controls. All samples were plated in triplicate, and values are expressed as the average of three independent experiments.

### 3.9. In Vitro Cytotoxicity and Anti-Biofilm Activity Assays

In vitro cytotoxicity assays were performed as described previously [12,24,25] with suitable modifications based on the type of bacteria. Cells were grown overnight in BHI/MHB broth at 37 °C and regrown in fresh medium for 24 h before centrifugation and suspension in deionized water. A cell suspension consisting of 10^6^ cells/mL was incubated with various concentrations of AgNPs for 24 h at 37 °C. After incubation, the bacteria were harvested at the indicated time points, and 100 μL aliquots were taken from each sample to determine the number of cfus. The experiment was performed with various controls, including a positive control (AgNPs and media without inoculum) and a negative control (media and inoculum without AgNPs). All samples were plated in triplicate, and values were averaged from three independent experiments.

Inhibition of biofilm formation was determined as described previously [12,25]. In brief, the cells were grown in BHI or MHB broth supplemented with 2% fetal calf serum, and individual wells of sterile, 96-well flat-bottom polystyrene tissue culture plates were filled with 180 μL of a single bacterial species (1 × 10^6^/mL). The cell culture plates were then incubated with AgNPs for 24 h at 37 °C. After incubation, the media were removed, and the wells were washed three times with 200 μL sterile distilled water to remove non-adherent bacteria. The crystal violet solutions in water were added for 45 min. The wells were then washed five times with 300 μL of sterile distilled water to remove excess stain. The absorbance of each well was measured at 595 nm using a microtiter ELISA reader. The percent inhibition of biofilm activity was calculated as described previously [12,25].

### 3.10. Measurement of LDH Activity 

LDH activity was determined by measuring the reduction of NAD^+^ to NADH and H^+^ during the oxidation of lactate to pyruvate according to a previously described method. Bacterial cells were adjusted to 10^6^ cfus/mL, and each culture was incubated in a shaking incubator at 37 °C for 12 h. AgNP concentrations were adjusted to the respective MIC concentration for each bacterium. After incubation with AgNPs, the culture was centrifuged at 4 °C for 30 min at 300× *g*, and the supernatant was discarded. The pellet was washed twice and then treated with LDH reaction solution in a microplate. The plate was then incubated with gentle shaking on an orbital shaker for 30 min at room temperature. After incubation, the OD of the plate was measured at 490 nm.

### 3.11. Measurement of ATP Levels

Measurement of ATP levels in the bacterial culture supernatant was conducted according to the method described previously [53]. In the luciferase-based assay, ATP levels were determined by measuring luminescence levels, which were compared against an ATP standard curve. In brief, 100 µL of the culture supernatant from the control or treated cells was mixed with an equal volume of BacTiter-Glo™ Microbial Cell Viability Assay Reagent in a 96-well opaque plate and incubated at room temperature for 5 min. After incubation, luminescence was read using a SpectraMax M2 plate reader (Molecular Devices, Sunnyvale, CA, USA).

### 3.12. Assay for the Leakage of Proteins and Reducing Sugars

Protein and sugar leakage from bacterial cells was determined as described previously [49,80,81]. In brief, the AgNP concentration was adjusted to the desired level for each isolate, and the concentration of bacterial cells was kept at 10^6^ CFUs/mL. Each culture was incubated in a shaking incubator at 37 °C for 4 h. Culture samples (1 mL) were centrifuged at 4 °C for 30 min at 10,000 rpm, and the supernatant was frozen at −20 °C before estimation of protein and sugar levels.

### 3.13. Measurement of ROS Levels

ROS generation was measured according to the previously described method using DCFDA [23,56]. Bacterial cells (10^6^ CFUs/mL) were treated with or without AgNPs at the required temperature for 12 h. After incubation, cells were centrifuged at 4 °C for 30 min at 300× *g*, after which the supernatant was treated with 100 μM DCFDA for 1 h. The amount of ROS produced in the sample was detected at the excitation wavelength of 485/20 nm and emission wavelength of 528/20 nm using a fluorescence multi-detection reader (BIOTEK, Winooski, VT, USA).

### 3.14. MDA Measurements

Cells grown in culture media were incubated at the required temperature, and MDA levels were determined using a thiobarbituric acid-reactive substances assay, as previously described, with suitable modifications [23,40,56]. In brief, 1 mL of culture medium of AgNP-treated cells was added to 10% sodium dodecyl sulfate and swirled vigorously. Subsequently, 2 mL of freshly prepared thiobarbituric acid was added to the mixture and incubated at 95 °C for 60 min. The reaction was allowed to cool at room temperature and centrifuged at 5000 rpm for 10 min. The OD of the supernatant was measured at 530 nm.

### 3.15. Measurement of Carbonylated Protein Content

The carbonylated protein content was measured according to a previously described method [82]. In brief, bacterial cells were treated with AgNPs for 12 h. The cells were harvested and then washed twice with phosphate buffer (pH 7, 0.05 M, 4 °C) by centrifugation at 5500× *g* for 20 min at 4 °C. The cells were resuspended in phosphate buffer and lysed by a sonicator. The protein carbonyl content was evaluated and expressed relative to the total protein content.

### 3.16. Measurement of NO

Bacterial cells were treated with AgNPs for 12 h, and then the NO production level was quantified spectrophotometrically using the Griess reagent (Sigma-Aldrich, St. Loius, MO, USA). The absorbance was measured at 540 nm and the nitrite concentration was determined using a calibration curve prepared with sodium nitrite as the standard [83].

### 3.17. Estimation of Antioxidants

For enzymatic determination, the cells were incubated at the required temperature with or without AgNPs for 12 h. The cells were pelleted by centrifugation at 10,000 rpm for 5 min, washed with PBS, and lysed (the lysate was prepared as described above). Oxidative stress markers, such as GSH, GSSG, SOD, catalase, and glutathione peroxidase activities were assayed with reagents from various kits, according to the respective instructions (Sigma-Aldrich).

### 3.18. Measurement of DNA/RNA Oxidation

DNA/RNA oxidation was measured according to a previously described method [22,55] along with manufacturer instructions. *P. melaninogenica* and *A. pyogenes* were treated with 0.8 and 1.0 μg/mL of AgNPs for 12 h, and then bacterial pellets were prepared as described previously [58]. DNA/RNA was purified according to the manufacturer instructions, and the 8-oxo-dG and 8-oxo-G levels were quantified.

### 3.19. Statistical Analysis

All experiments were carried out in triplicate and repeated at least three times. The results are presented as means ± standard deviations of three separate experiments. Statistically significant differences between treatment and control groups were determined using Student’s *t* test (*p* < 0.05).

## 4. Conclusions

The inadequate isolation, identification, and subsequent performance of susceptibility testing of bacteria from an infected site are possible reasons for the emergence of antimicrobial resistance. In particular, uterine infections are caused by aerobic, facultative anaerobic, and obligate anaerobic microorganisms, which are common causes of bacterial infections of endogenous origin. Failure to develop suitable therapy against bacteria often leads to clinical failures. We explored the possibility of nanoparticle-mediated therapy as a new strategy to fight against uterine infections in dairy cattle by the isolation and identification of MDR pathogens, such as *P. melaninogenica* and *A. pyogenes*, from cow uterine samples. To develop a new antimicrobial therapy against antibiotics, we synthesized AgNPs with an average size of 10 nm using a novel biomolecule called apigenin, a flavone class compound. The synthesized AgNPs exhibited significant antibacterial and anti-biofilm activity against representative Gram-negative and Gram-positive bacteria, such as *P. melaninogenica* and *A. pyogenes.* Nevertheless, the specific mechanism of the antibacterial effect of AgNPs against *P. melaninogenica* and *A. pyogenes* remains unknown. This is the first study to suggest that the mechanism of cell death of AgNPs is mainly related to oxidative and nitro-oxidative stress induced by the generation of ROS, which results in increased leakage of LDH, proteins, and sugars, along with the depletion of ATP, with simultaneous increases in the level of MDA, the protein carbonyl content, and NO in *P. melaninogenica* and *A. pyogenes*. The alarming rate of oxidative stress increase leads to an imbalance of antioxidant levels, which eventually leads to the oxidation of macromolecules, such as DNA and RNA, under exposure of bacteria to AgNPs. This work shows that biomolecule-assisted AgNPs are efficient, stable, and biocompatible without any undesired side effects against pathogenic bacteria causing metritis and endometritis. This novel formulation and insight into how AgNPs impact bacterial metabolism could provide key information on the mechanisms of action, leading to enhanced therapeutic methodologies for the development of better antibacterial agents to benefit humans and domestic animals.
